# Case report: Cefoperazone-sulbactam induced Kounis syndrome and cardiogenic shock

**DOI:** 10.3389/fcvm.2022.1061586

**Published:** 2022-11-16

**Authors:** Peng Ding, Yuan Zhou, Kun-lan Long, Li Zhang, Pei-yang Gao

**Affiliations:** ^1^Department of Critical Care Medicine, Hospital of Chengdu University of Traditional Chinese Medicine, Chengdu, China; ^2^Department of Geriatric Medicine, General Hospital of Western Theater Command, PLA, Chengdu, China; ^3^Department of Cardiology, Hospital of Chengdu University of Traditional Chinese Medicine, Chengdu, China

**Keywords:** Kounis syndrome, cefoperazone-sulbactam, cardiogenic shock, allergy, intra-stent thrombosis, case report

## Abstract

**Background:**

Kounis syndrome is a hypersensitive coronary artery disease caused by the body's exposure to allergens, which is induced by various drugs and environmental factors. This entity has been described primarily in isolated case reports and case series. We report a case of type III Kounis syndrome caused by cefoperazone-sulbactam.

**Case presentation:**

A 79-year-old man who received an infusion of cefoperazone-sulbactam in Respiratory Department of our hospital for recurrent infections. 28 minutes later, he developed skin flushing of the trunk and extremities, soon followed by loss of consciousness and shock. With antianaphylaxis, pressor therapy, and fluid rehydration, the patient was admitted to the ICU for treatment. During which, he experienced recurrent ventricular fibrillation and a progressive increase in troponin I levels. The ECG of the patient showed that the ST segment elevation of lead II, III, avF, and V3R–V5R was 0.10–0.20 MV. An urgent coronary angiography showed an in-stent thrombosis in the middle part of the right coronary artery, occlusion of the distal flow with TIMI grade 0. The diagnosis was type III Kounis syndrome with cardiogenic shock. Despite aggressive treatment, the patient died on day 7 after ICU admission.

**Conclusion:**

Kunis syndrome is a life-threatening disease, and therefore allergic reactions in patients with a history of cephalosporin allergy and coronary stent implantation should be considered and treated promptly.

## Introduction

Kounis syndrome has been defined as a coronary syndrome resulting from interaction with mast cells and inflammatory cells, which in turn induces a hypersensitivity response. It is not only a single organ disease, but also a complex disease involving multiple systems, with a significant impact on morbidity and mortality (1). As its diagnosis is extremely dependent on interventional imaging, it is difficult for clinicians to make an accurate diagnosis in a short time, which will lead to serious consequences. Given the serious adverse effects associated with cephalosporin infusion, clinicians should consider Kounis syndrome in every patient receiving cephalosporin therapy who presents with acute chest pain or allergic symptoms (2). Antibiotic associated Kounis syndrome has been reported, the most common being cefazolin (3), cefuroxime (4), and amoxicillin (5), whereas cefoperazone-sulbactam are rarely reported. Here, we report a case of type III Kounis syndrome induced by cefoperazone-sulbactam.

## Case presentation

A 79-year-old male patient was admitted to the Respiratory Department of our hospital on August 15, 2022, because of fever, cough, sputum production and dyspnea after cold. On July 8, 2022, the patient underwent coronary angiography in the Cardiology Department of our hospital due to coronary artery disease, and a stent was implanted in the right coronary artery. The patient regularly took aspirin enteric-coated tablets, clopidogrel bisulfate tablets and atorvastatin calcium tablets. Other past medical conditions included hypertension, atrial fibrillation, and tuberculosis. The patient's allergic history included iodine (allergic reaction was local skin erythema papules) and “cephalosporins” (allergic reaction was urticaria in the limbs), but the patient had been repeatedly infused with cefazoxime, cefpilamine, ceftazidime and other drugs, no allergic reaction occurred. On the morning of August 23, 28 minutes after the injection of cefoperazone-sulbactam, the patient developed numbness in the extremities, chest tightness, palpitations, flushing of the skin in the extremities and body, progressive loss of consciousness, wheezing, sweating, and cyanosis of the lips. The bedside ECG monitor showed: heart rate of 80 beats per minute, with blood pressure and oxygen saturation being undetectable. The respiratory physician considered that the patient had developed severe anaphylactic shock. Then, them immediately stopped the suspicious fluid infusion, opened the airway, administered epinephrine 1 mg (again 1 mg after 5 minutes), with rapid infusion of sodium chloride injection 1,000 ml and methylprednisolone sodium succinate 40 mg. ICU physicians immediately established central venous access and tracheal intubation upon arrival, maintained blood pressure with norepinephrine and epinephrine, and assisted the patient's breathing with an invasive ventilator. The patient was then transferred to the ICU on advanced life support.

**Figure 1:**
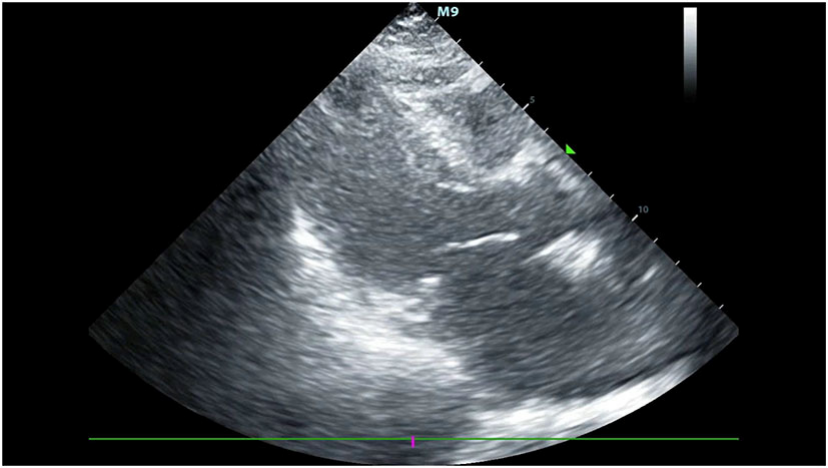
The bedside cardiac ultrasound suggested poor cardiac contractile function and left ventricular ejection fraction only 25%.

**Figure 2:**
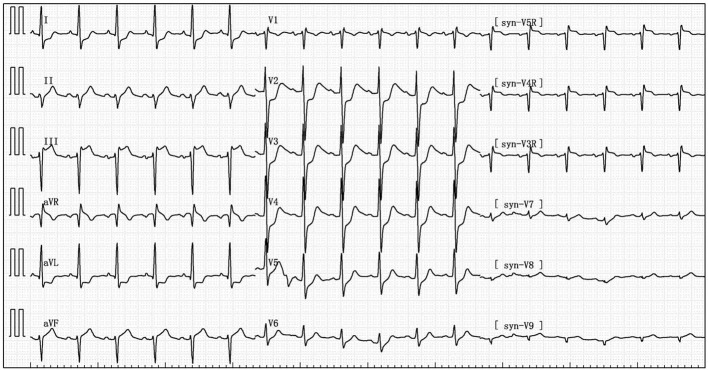
The after admission to ICU, 18-lead electrocardiogram (ECG) showed that the ST segment of lead I, aVL, V2–V6 depression 0.1–0.3 mV, and the ST segment of lead II, III, avF, V3R-V5R elevation 0.10–0.20 mV.

**Figure 3:**
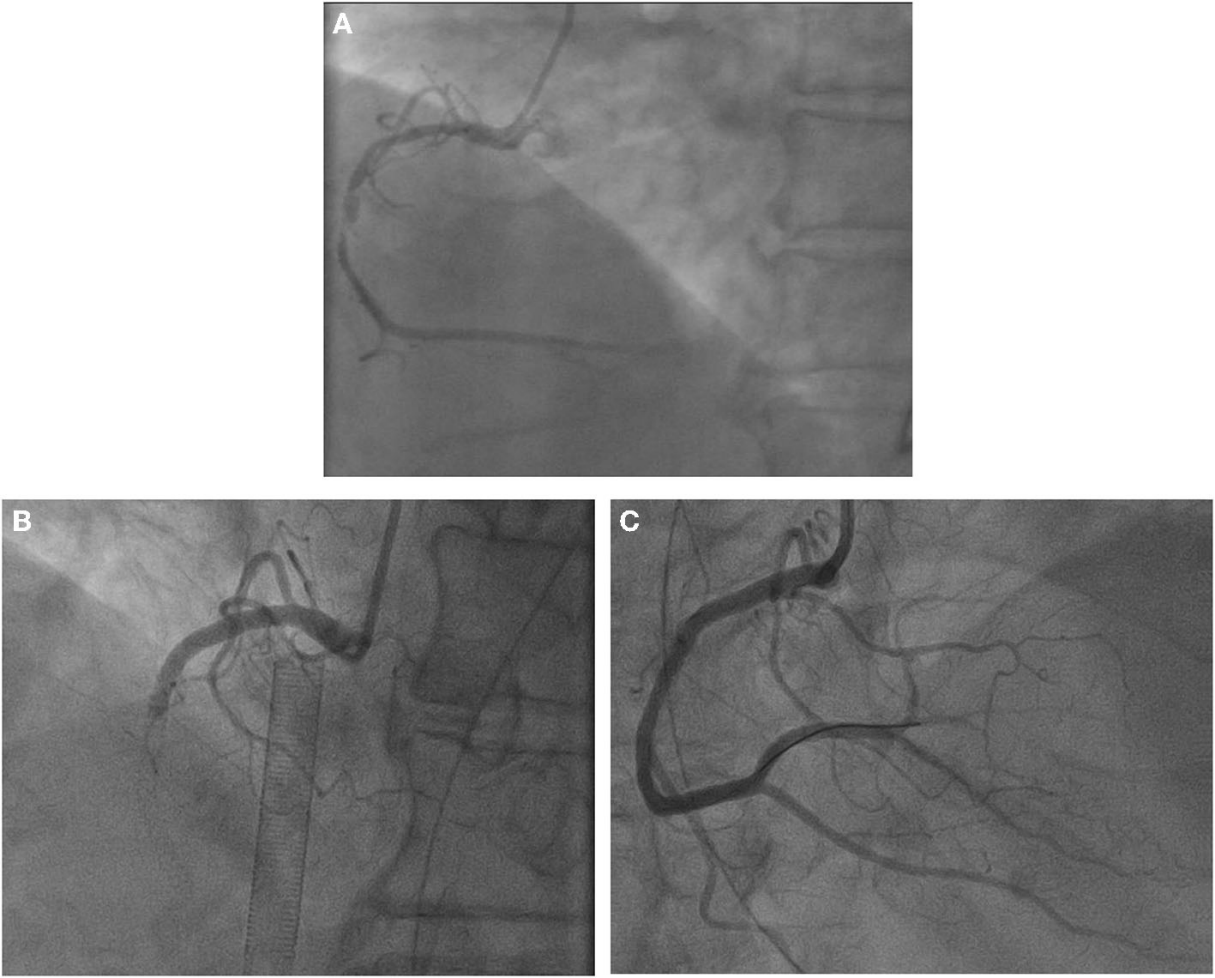
**(A)** Right coronary angiography 1 months ago. Results of coronary angiography. **(B)** Right coronary artery: stent shadow was seen from the proximal segment to the middle segment, complete occlusion in the middle stent, myocardial infarction (TIMI) blood flow grade 0. **(C)** Coronary angiography after balloon dilation and thrombus aspiration showed no stenosis, and TIMI flow was grade 3.

**Figure 4:**
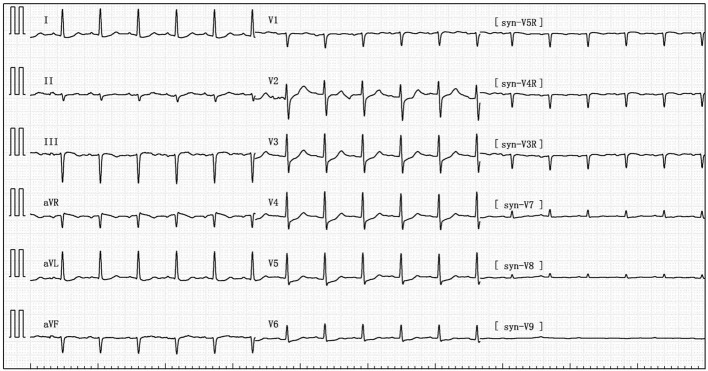
Postoperative electrocardiogram showing ST segment elevation 0.05–0.1 MV in lead III and avF.

Her blood pressure on arrival at the ICU was 92/52 mmHg (at this point the norepinephrine and epinephrine doses were 1.25 and 0.83 μg/kg/min, respectively), but the patient was extremely hemodynamically unstable, and bedside cardiac ultrasound suggested poor cardiac contractile function and left ventricular ejection fraction only 25% (Figure 1). What's worse, the patient's norepinephrine dose was increased to 3.5 μg/kg/min, and ventricular fibrillation occurred frequently. The ICU physician immediately treated the patient with veno-arterial extracorporeal membrane oxygenation (V-A ECMO) and intra-aortic balloon pump (IABP). After admission to ICU, 18-lead electrocardiogram (ECG) showed a new left anterior fascicular block, the ST segment of lead I, aVL, V2–V6 depression 0.1–0.3 mV, and the ST segment of lead II, III, avF, V3R–V5R elevation 0.10–0.20 mV (Figure 2). Serum cardiac troponin I was 22.35 ng/mL (normal range 0–0.05). Upon completion of our consultation with the cardiologist, the patient was considered to have had an acute myocardial infarction, which required urgent percutaneous coronary angiography (the right coronary angiography results 1 months earlier was shown in Figure 3A). The results showed stent shadow in the proximal and middle segments of the right coronary artery, mild intimal hyperplasia in the stent, complete occlusion in the middle, and myocardial infarction (TIMI) flow of grade 0 (Figure 3B). Right coronary artery lesions were pretreated with 2.0 mm × 20 mm pre-dilated balloon and 3.0 mm × 10 mm open balloon, and then percutaneous transluminal coronary angioplasty was performed with 3.5 mm × 30 mm balloon into the stent. Re-angiography showed no stenosis in the right coronary stent and TIMI flow of grade 3 (Figure 3C). The patient was sent back to ICU after completion of surgery. By then, the postoperative ECG showed that ST-segment elevation of lead III and avF was 0.05–0.1 MV (Figure 4). Laboratory tests suggested a serum immunoglobulin E concentration of 290 IU/ml (normal range 0–165). The diagnosis of type III Kounis syndrome with cardiogenic shock was made, and the condition manifested as acute ST-segment elevation myocardial infarction caused by allergic stent thrombosis.

After from percutaneous coronary intervention, the patient continued to receive ECMO combined with IABP, norepinephrine, epinephrine and other drugs. However, the patient's heart function did not recover and he died seven days later. Cause of death: cardiogenic shock.

## Discussion and literature review

Kounis syndrome is an acute coronary syndrome of allergic reaction-mediated coronary artery spasm with or without erosion or rupture of atherosclerotic plaques. Kounis syndrome should be suspected when patients present with symptoms such as chest pain and dyspnea and generalized allergic reactions (e.g., red rash and wheezing), even in the absence of a history of coronary heart disease (6). Allergic coronary artery spasm, allergic myocardial infarction, and in-stent thrombosis are the three types of this syndrome. The central pathogenesis is the activation of mast cells due to an allergic reaction, which leads to a massive release of pro-inflammatory mediators (including histamine, tryptophanase, leukotrienes, platelet-activating factor, and heparin), resulting in coronary spasm, plaque rupture, and thrombosis (7). A variety of medications, food and environmental factors can trigger this, with the most common triggers are antibiotics (27.4%) and insect bites (23.4%) (8). History of specific medications, occurrence of allergic reactions and cardiovascular system symptoms are the main diagnostic basis for Kounis syndrome. Serum tryptophan, histamine, cardiac enzymes, cardiac troponin, trypsin, electrocardiogram, echocardiogram, and angiography help to make the correct diagnosis (9). Therefore, clinicians should have a detailed understanding of the patient's pathogenesis perform a complete physical examination, and take targeted tests to achieve a rapid and accurate diagnosis of Kounis syndrome.

More than 70 years since 1950, Pfister et al. (10) reported the first case of myocardial infarction and urticaria after penicillin administration, but Kounis syndrome still could be found as case reports. This suggests its low incidence. In the study by Abdelghany et al. (8), 175 patients with Kounis syndrome developed cardiogenic shock in 2.3% and mortality was 2.9%. Throughout the research, Dai et al. (11) found that Kounis syndrome occurred within 20 minutes after exposure to predisposing factors in perioperative patients. Recently, a large epidemiological study (12) from the United States reported 235,420 patients hospitalized for anaphylaxis/hypersensitivity/anaphylaxis. 2,616 patients (1.1%) developed an acute coronary syndrome and were diagnosed with Kounis syndrome, and subsequent in-hospital mortality from any cause was 7.0% (OR: 9.74, 95% CI: 8.08–11.76). With the introduction of COVID-19 vaccines, adverse cardiac events related to COVID-19 continue to emerge. Approximately 77 patients have been reported, 35 of whom had acute myocardial infarction (which could be diagnosed as Kounis syndrome) and 42 had myocarditis (13). With the increased popularity of this disease, its clinical diagnosis rate will increase as well.

Cefoperazone-sulbactam is a third-generation cephalosporin (cefoperazone) combined with β-lactamase inhibitor (sulbactam). It has broad spectrum antibacterial activity and is used to treat various types of infections (such as nosocomial pneumonia, intraperitoneal infection, gynecological infection, sepsis, etc.) (14). Cephalosporin itself does not cause allergic reactions. Only when it is combined with macromolecular substances such as proteins or peptides in the body or polymerized into macromolecular impurities in the process of production, storage and use of cephalosporin can it become a full antigen and induce the body to produce IgE antibody, thus leading to rapid allergic reactions. Studies have shown that β-lactam allergy is mediated by the specific antigenic determinant IgE, which is based on the whole β-lactam and protein carrier molecules, and the differences between the class-specific side chains and the R1 and R2 side chains provide antigenic specificity (15). For patients with allergic reactions, skin test has a very high diagnostic value (16). In this case, although the patient had a history of “cephalosporin” allergy, multiple cephalosporins had been infused during the previous hospitalization and no allergic reaction had occurred, so a definitive diagnosis could not be obtained on the skin test. The patient developed wheezing, chest tightness, and skin flushing after an intravenous infusion of cefoperazone-sulbactam. The patient was diagnosed with cefoperazone-sulbactam induced type III Kounis syndrome based on his history of allergy and medication, electrocardiogram, laboratory tests (troponin I and immunoglobulin E), and coronary angiography.

There is no standardized treatment plan for the treatment of Kounis syndrome. Most clinicians will take antiallergy and antiplatelet therapy, relieve vasospasm, perform coronary angiography and open the vessel as soon as possible (17). Epinephrine is the first-line treatment for anaphylactic shock, and second-line drugs include antihistamines or glucocorticoids. However, the use of epinephrine may cause coronary artery contraction or malignant arrhythmia, and its benefits and potential harms should be carefully considered in clinical use (18). The effect of short-term glucocorticoid infusion is ideal and relatively safe. Antihistamines have the side effect of hypotension, which is not suitable for patients with anaphylactic shock (19). Vasodilators, such as nitrate and calcium channel blockers, can relieve allergic vasospasm but may induce and worsen hypotension (6). Therefore, clinicians have to be careful to select appropriate and safe treatment options.

This case report has the following limitations. First, it is not possible to draw a general conclusion only by showing the patient's medical experience. Second, due to the limitation of conditions, pulmonary artery catheter monitoring was not performed, and the cardiac function index of patients could not be accurately obtained. Finally, the family's withdrawal of treatment can have a significant impact on the patient's final outcome. However, our discovery of a rare type III Kounis syndrome induced by cefoperazone-sulbactam is its strengths.

## Conclusion

Kounis syndrome is an indiscernibly and life-threatening condition. Patients with a history of both cephalosporin allergy and intracoronary stent implantation who develop allergic reactions should be considered quickly and given prompt treatment.

## Data availability statement

The original contributions presented in the study are included in the article/supplementary material, further inquiries can be directed to the corresponding author.

## Ethics statement

Written informed consent was obtained from the individual(s) for the publication of any potentially identifiable images or data included in this article.

## Author contributions

PD and YZ drafted the manuscript and are credited as co-first authors. K-lL and PD provided clinical specimens and information. LZ provided the figures. P-yG revised the manuscript. All authors read and approved the final manuscript.

## Funding

This work was supported by the National Natural Science Foundation of China (No. 81873298).

## Conflict of interest

The authors declare that the research was conducted in the absence of any commercial or financial relationships that could be construed as a potential conflict of interest.

## Publisher's note

All claims expressed in this article are solely those of the authors and do not necessarily represent those of their affiliated organizations, or those of the publisher, the editors and the reviewers. Any product that may be evaluated in this article, or claim that may be made by its manufacturer, is not guaranteed or endorsed by the publisher.

## References

[1] Kounis NG Koniari I Velissaris D Tzanis G Hahalis G. Kounis Syndrome—not a single-organ arterial disorder but a multisystem and multidisciplinary disease. Balkan Med J. (2019) 36:212–21. 10.4274/balkanmedj.galenos.2019.2019.5.62 31198019 PMC6636655

[2] Fang W Song L Deng Z Sun W Li Z Wang C. Analysis of clinical features of Kounis syndrome induced by cephalosporin. Front Cardiovasc Med. (2022) 9:885438. 10.3389/fcvm.2022.885438 35557534 PMC9086825

[3] Adachi H Ihara M Nojima Y Kurimoto T Nanto S. Kounis syndrome caused by anaphylaxis without skin manifestations after cefazolin administration. J Allergy Clin Immunol Pract. (2019) 7:317–9. 10.1016/j.jaip.2018.05.030 29902529

[4] Absmaier M Biedermann T Brockow K. Allergic myocardial infarction (Kounis syndrome) after cefuroxime with side-chain cross-reactivity. J Allergy Clin Immunol Pract. (2018) 6:1781–3.e1. 10.1016/j.jaip.2018.01.033 29428243

[5] Pradhan S Christ M Trappe HJ. Kounis syndrome induced by amoxicillin following vasospastic coronary event in a 22-year-old patient: a case report. Cardiovasc Diagn Ther. (2018) 8:180–5. 10.21037/cdt.2018.03.07 29850410 PMC5951989

[6] Wu HY Gao TJ Cao YW You PH. Case report: phloroglucinol-induced Kounis syndrome. Front Cardiovasc Med. (2021) 8:668318. 10.3389/fcvm.2021.668318 34012985 PMC8126616

[7] Fassio F Losappio L Antolin-Amerigo D Peveri S Pala G Preziosi D. Kounis syndrome: a concise review with focus on management. Eur J Intern Med. (2016) 30:7–10. 10.1016/j.ejim.2015.12.004 26795552

[8] Abdelghany M Subedi R Shah S Kozman H. Kounis syndrome: a review article on epidemiology, diagnostic findings, management and complications of allergic acute coronary syndrome. Int J Cardiol. (2017) 232:1–4. 10.1016/j.ijcard.2017.01.124 28153536

[9] Kounis NG. Kounis syndrome: an update on epidemiology, pathogenesis, diagnosis and therapeutic management. Clin Chem Lab Med. (2016) 54:1545–59. 10.1515/cclm-2016-0010 26966931

[10] Pfister CW Plice SG. Acute myocardial infarction during a prolonged allergic reaction to penicillin. Am Heart J. (1950) 40:945–7. 10.1016/0002-8703(50)90191-8 14789736

[11] Dai B Cavaye J Judd M Beuth J Iswariah H Gurunathan U. Perioperative presentations of Kounis syndrome: a systematic literature review. J Cardiothorac Vasc Anesth. (2022) 36:2070–6. 10.1053/j.jvca.2022.01.042 35260322

[12] Desai R Parekh T Patel U Fong HK Samani S Patel C. Epidemiology of acute coronary syndrome co-existent with allergic/hypersensitivity/anaphylactic reactions (Kounis syndrome) in the United States: a nationwide inpatient analysis. Int J Cardiol. (2019) 292:35–8. 10.1016/j.ijcard.2019.06.002 31204069

[13] Aye YN Mai AS Zhang A Lim OZH Lin N Ng CH. Acute myocardial infarction and myocarditis following COVID-19 vaccination. QJM. (2021) hcab252. 10.1093/qjmed/hcab252 34586408 PMC8522388

[14] Sader HS Carvalhaes CG Streit JM Castanheira M Flamm RK. Antimicrobial activity of cefoperazone-sulbactam tested against Gram-Negative organisms from Europe, Asia-Pacific, and Latin America. Int J Infect Dis. (2020) 91:32–7. 10.1016/j.ijid.2019.11.006 31715325

[15] Khan DA Banerji A Bernstein JA Bilgicer B Blumenthal K Castells M. Cephalosporin allergy: current understanding and future challenges. J Allergy Clin Immunol Pract. (2019) 7:2105–14. 10.1016/j.jaip.2019.06.001 31495420 PMC6955146

[16] Al-Ahmad M Edin J Musa F Rodriguez-Bouza T. Drug allergy profile from a national drug allergy registry. Front Pharmacol. (2021) 11:555666. 10.3389/fphar.2020.555666 33542684 PMC7851708

[17] Tan PZ Chew NWS Tay SH Chang P. The allergic myocardial infarction dilemma: is it the anaphylaxis or the epinephrine? J Thromb Thrombolysis. (2021) 52:941–8. 10.1007/s11239-021-02389-4 33544285

[18] Lieberman P Simons FE. Anaphylaxis and cardiovascular disease: therapeutic dilemmas. Clin Exp Allergy. (2015) 45:1288–95. 10.1111/cea.12520 25711241

[19] Fein MN Fischer DA O'Keefe AW Sussman GL. CSACI position statement: newer generation H1-antihistamines are safer than first-generation H1-antihistamines and should be the first-line antihistamines for the treatment of allergic rhinitis and urticaria. Allergy Asthma Clin Immunol. (2019) 15:61. 10.1186/s13223-019-0375-9 31582993 PMC6771107

